# Post-Chromatographic Derivatization Coupled with Mass Spectrometry as a Method of Profiling and Identification of Antioxidants; *Ligustrum vulgare* Phytocomplex as an Example

**DOI:** 10.3390/molecules28248000

**Published:** 2023-12-07

**Authors:** Szymon Litewski, Marika Mróz, Agnieszka Bartoszek, Barbara Kusznierewicz

**Affiliations:** Department of Chemistry, Technology and Biotechnology of Food, Faculty of Chemistry, Gdańsk University of Technology, 11/12 Narutowicza St., 80-233 Gdańsk, Poland; szymon.litewski@pg.edu.pl (S.L.); marika.mroz@pg.edu.pl (M.M.)

**Keywords:** antioxidants, *Ligustrum vulgare*, HPTLC, HPLC, HRMS, ABTS, DPPH

## Abstract

High-performance liquid chromatography (HPLC) and high-performance thin-layer chromatography (HPTLC) coupled with radical scavenging assays, such as 2,2-diphenyl-1-picrylhydrazyl (DPPH) and 2,2′-azinobis-(3-ethylbenzothiazoline-6-sulfonic acid (ABTS) can be both used for the detection of the antioxidants in plant extracts. In this study, the ethanolic (70% *v*/*v*) extracts from different morphological parts of *Ligustrum vulgare* collected at different stages of maturity were used as the source of antioxidants. The final identification of antioxidants was performed using high-resolution mass spectroscopy (HRMS). As a result, 19 compounds with antioxidant properties detected with HPLC-ABTS assay and 10 compounds detected with HPTLC-DPPH/ABTS assay were identified, mostly from the group of iridoids, phenylethanoids, and flavonoids. When comparing different *L. vulgare* samples, it was found that the extracts obtained from leaves contained the greatest number of antioxidants. The results of this study suggest that HPTLC-DPPH/ABTS as well as HPLC-ABTS derivatization coupled with the HRMS can be successfully used for profiling and identification of antioxidants from natural sources. Planar chromatography is more suitable for screening multiple samples because of its simplicity, whereas more challenging liquid chromatography provides more detailed information and is therefore better for a selected set of samples.

## 1. Introduction

Plant secondary metabolites have been of interest for ages, because of their diverse biological activities, important in nutrition and medicine. Nonetheless, despite continuous international research efforts, the majority of the phytomes of earth’s plants remain unknown, both as regards chemical composition and, what is even more important, biological properties. Therefore, the analytical strategies that could accelerate the characterization of plant bioactives are badly needed. Regardless of the great variety of sources, chemical structures, and biological activities, a large proportion of phytochemicals often share one common chemical property, which is the low reductive potential. This property could thus be exploited as a marker of bioactivity in the first screenings of phytomes.

Total antioxidant activity determination is useful in the preliminary characterization of plant material as it indicates which samples may become those of particular interest in the search for rich sources of bioactive compounds. There are several simple analytical methods based on different mechanisms to measure the total antioxidant activity of a sample. They are often based on redox reactions and assess either their kinetics or the point of reaching the equilibrium state. These chemical reactions involving antioxidants present in samples usually lead to the appearance of characteristic colours or discoloration of the prooxidant solutions, which is monitored spectrophotometrically at a specific maximum absorbance wavelength [1]. Typically, the total antioxidant capacity of the sample is expressed as EC_50_ value, i.e., the concentration necessary to reduce 50% of radicals, or by the Trolox equivalent antioxidant capacity (TEAC) index. However, approaches of this kind are associated with various well-known limitations, such as dependence on the initial radical prooxidant concentration and the limited chemical specificity towards antioxidants. Among these methods, there are two assays, namely 2,2′-azinobis-(3-ethylbenzothiazoline-6-sulfonic acid (ABTS) test and the [2,2-di(4-tert-octylphenyl)-1-picrylhydrazyl] (DPPH) test, that are based on the transfer of both a hydrogen atom and an electron and indicate specifically antiradical activity of the sample. Both approaches apply to pure compounds as well as plant-derived extracts, as presented by Baranowska et al. (2018) [2] and Kusznierewicz et al. (2021) [3]. More importantly, the incorporation of reaction kinetics into antioxidant activity assessment in cell-free systems seems to be better correlated with reducing properties of substances assessed in biological systems by e.g., cellular antioxidant activity assay [4].

Although the chemical assays provide basic information about beneficial properties, they omit two important factors: the number and type of antioxidants present in the sample. Another limitation of total antioxidant activity assays is that the antioxidants react differently in each assay. Furthermore, the major restriction in the case of in vitro results is that actual in vivo antioxidant performance may vary dramatically from in vitro activity. Additionally, the final impact of antioxidants on human organisms may differ when they are ingested/administered in combination with other chemical components of food/drugs/supplements which can either interfere or strengthen the expected effect. It should be stressed, though, that all batch methods employ radicals/oxidants with no physiological relevance, which is their major limitation. Nonetheless, they have several advantages, being inexpensive, quick, and easy to perform, and in many situations, such as in food production, they are sufficient to compare antioxidant properties between different samples. Therefore, at present, the assessment of antioxidant activity should be considered as a convenient method for identifying reducing substances in plant and food samples, rather than as a means of assessing their health benefits. 

Nevertheless, as already mentioned many reducing phytochemicals are indeed valuable bioactive compounds; therefore, their detection in plant raw materials and their monitoring in the food or dietary supplement production chain are increasingly recognized as an important issue. However, in this case, the determination of the total antioxidant activity does not provide satisfactory information on the composition of antioxidants in the plant raw material nor their possible transformations during processing. Therefore, at this point, the use of tools that enable obtaining and characterizing the antioxidant profile is more desirable. One such approach can be based on high-performance liquid chromatography (HPLC). Firstly, this technique by monitoring chromatographic separation at multiple wavelengths simultaneously allows not only to detection of analytes with very different absorption spectra but also provides initial clues as regards their identity. Secondly, if appropriate standards are available, quantitative analysis based on registered peak areas is possible. Most importantly, detected substances might be subjected to further analysis in a modified HPLC system to indicate those that exhibit antiradical activity. Since 1999, there have been reports in the literature suggesting the possibility of using an HPLC system for online detection of antioxidants in the effluent from a column after derivatization with a suitable reagent [5]. Since then, there have been regular articles on the use of luminol, ABTS, and DPPH radicals and Folin–Ciocalteu reagent for the derivatization of analytes in HPLC eluate [6,7,8,9,10,11]. In the most studied approach, a DPPH or ABTS radical solution is added post-column to the HPLC flow. The basis for the detection of antioxidants present in a sample is the decrease in absorbance in the visible wavelength range as a result of the conversion of these radicals to their colorless, reduced forms. Post-column addition of the indicator can be achieved using a “homemade” kit, usually consisting of reaction coils made of PEEK tubes powered by an additional pump. Another, more convenient, solution is the use of commercially available post-column derivatizers [12,13]. Such an approach grants not only the effective separation of individual compounds but also ensures a confident indication of redox-active substances. Moreover, it provides numerical data for calculating the antioxidant activity of individual compounds and their contribution to the total antioxidant activity of the analyzed sample. 

Apart from HPLC, thin-layer chromatography (TLC), a simple and cost-effective technique, is commonly used in studies on natural products. TLC separation on the one hand reveals the overall complexity of samples, not limited by the method of detection, on the other hand enables the quantitative assessment of separated analytes [3]. Another extension of this technique is TLC bioautography combining planar chromatographic separation and biological activity detection. In the case of antioxidant activity, DPPH [14,15], ABTS [14], and β-carotene [16] reagents are most used to detect the reducing agents resolved on the plate. A modified, improved, and automated version of TLC in the form of high-performance thin-layer chromatography (HPTLC) has proven to be particularly useful for screening and fingerprinting of bioactives, due to the vast possibilities and simplicity of in situ derivatization, or conducting bioassays, both before and after chromatogram development. Therefore, HPTLC-based techniques are of choice in comparative studies, because they enable quick assessment of the presence of antioxidants in a sample. Furthermore, the advanced HPTLC system allows for tentative qualitative analyses based on image documentation and densitometric quantification of separated compounds. 

The possibility of obtaining such “fingerprints” of antioxidant compounds using the HPLC and HPTLC methods found in plant material provides important information, complementing spectrophotometric tests, which compounds and to what extent influence the antioxidant properties of the sample. Furthermore, when combined with spectroscopic methods such as mass spectrometry (MS), based on antioxidant profiles, the identification of structures of analytes exhibiting antioxidant properties becomes possible.

Antioxidant profiling, combined with parallel MS analysis used for compound identification, provides the most detailed information on the composition of reducing agents, which turned out to be extremely useful in studies on natural products, in particular when characterizing various plant matrices, e.g., fruits [15,17,18,19], vegetables [3], herbs [20,21,22,23,24] or beverages [25,26]. This approach is also used for monitoring changes in the phytocomplexes of plants exposed to the influence of various factors. For example, the comparative analyses of chromatographic profiles of antioxidants enabled the identification of the most labile antioxidants that are degraded during high-temperature processing such as pasteurization or roasting as well as the detection of new compounds formed during these processes, which also displayed antioxidant activity [27,28]. The post-column derivatization method also turned out to be very useful in studies on the impact of various factors such as extraction conditions [21], cultivation conditions [19], or post-harvest treatment [29] on the antioxidant profile of various plant raw materials. The advent of new equipment solutions in the form of a TLC-MS interface made it possible to directly analyze zones from the TLC and HPTLC plates by mass spectrometry. This solution is gaining popularity in the identification of various compounds, including antioxidants. As an example, may serve the research where initial detection using a DPPH indicator and subsequent MS analysis of analytes from selected zones of the TLC plate allowed the identification of antioxidant phenolics in *Cyclanthera pedata*, *Genista saharae,* or propolis [30,31,32].

This study aimed to demonstrate and compare the usefulness of two different high-performance chromatographic techniques, namely HPLC and HPTLC, combined with redox reactions and coupled with mass spectrometry, for the separation and identification of plant antioxidants. Two commonly used reagents, namely ABTS and DPPH, were used as indicators of antioxidant compounds. The effectiveness of both techniques, HPLC/antioxidants profiling/MS and HPTLC/antioxidants profiling/MS was compared using the example samples derived from the same plant matrix, i.e., extracts from different morphological parts of common privet (*Ligustrum vulgare*) in different stages of maturity. *L. vulgare* is a plant from the *Oleaceae* family, which is characteristic of Southeast Asia and is an integral part of Chinese folk medicine. Although *L. vulgare* is a known source of oleuropein, a powerful antioxidant agent, no such comprehensive studies have been carried out to date that consider other antioxidants contained in this plant.

## 2. Results and Discussion

### 2.1. Antioxidants Separation and Identification by HPTLC-MS

**Figure 1:**
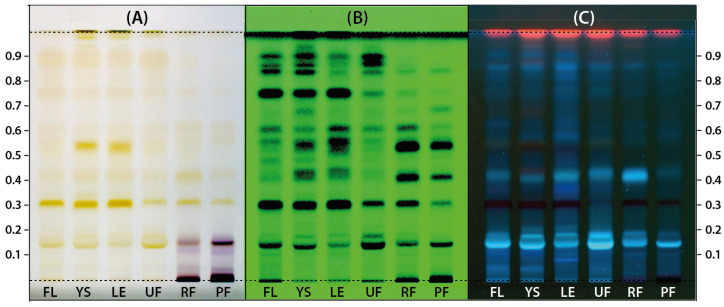
The HPTLC chromatograms of extracts prepared from *Ligustrum vulgare* flowers (FL), young shoots (YS), leaves (LE), and unripe (UF), ripe (RF) and postseason (PF) fruits photographed under white light, (**A**) at 254 nm (**B**) and 366 nm (**C**).

Different morphological parts of *Ligustrum vulgare* (LV) collected at different stages of the growing season were extracted using a mixture of ethanol and water which is regarded as a low-toxicity extractant. Profiles of phytochemicals present in these extracts were prepared using the HPTLC technique according to the methodology dedicated to the determination of compounds belonging to secoiridoids and phenylpropanoids as proposed by Czerwińska et al. 2015 [33]. Only a slight modification in the composition of the mobile phase was introduced by adding a larger amount of formic acid. Increasing the content of formic acid allowed for the achievement of better separation of individual compounds because of the higher density and polarity of the mobile phase. The chromatograms obtained at this stage were photographed under white light as well as at 254 and 366 nm for visualization of different compounds and are presented in Figure 1. HPTLC profiles recorded under white light (Figure 1A) indicate the presence of mostly the same compounds at various concentration levels in all morphological parts of the common privet. However, several purple bands were detected in ripe fruits (RF) and post-season fruits (PF) that were not present in other samples and most likely originated from anthocyanins, which, according to the literature data on *L. vulgare*, are mainly glycosidic forms of delphinidin, pelargonidin, and cyanidin [34]. Images of chromatograms taken at 254 and 366 nm (Figure 1B,C) also indicate the similarity of the composition of the extracts, again with some differences in the concentration levels of several compounds.

**Figure 2:**
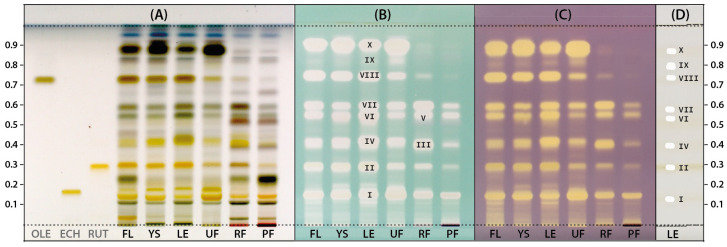
The HPTLC chromatograms of extracts prepared from *Ligustrum vulgare* flowers (FL), young shoots (YS), leaves (LE) and unripe (UF), ripe (RF) and postseason (PF) fruits derivatized with vanillin (**A**), ABTS (**B**) and DPPH (**C**) reagent juxtaposed with an example track showing isolated zones with antioxidants subjected to MS analysis (**D**). All plates were photographed under white light. The compound numbers (I–X) correspond to the numbers in Table 1.

To obtain more detailed profiles, the HPTLC plate was sprayed with vanillin reagent. According to Do et al. (2021) [35], the vanillin reagent may be a suitable alternative to the anisaldehyde reagent, which is widely used to detect various natural products whose native forms are not colored. Chromatograms obtained for the LV extracts and three additional solutions of the standards: oleuropein (OLE), echinacoside (ECH), and rutin (RUT) derivatized with the vanillin reagent were photographed in white light and are presented in Figure 2A. The mentioned reference compounds were selected due to their documented presence in *Ligustrum* species and their confirmed antioxidant properties. Their presence was also confirmed in the tested LV extracts based on the consistency of color and Rf parameters of the appropriate bands. In the case of OLE and RUT, the most intense bands indicating their high concentration were observed for extracts from flowers, leaves, and young shoots of LV. In turn, the extract from unripe fruit contained the highest ECH amount.

To obtain antioxidant profiles, the next two HPTLC plates were developed under the same conditions as previously and then sprayed with methanol solutions of either ABTS or DPPH radicals (Figure 2B,C). The use of solutions of these radicals as derivatizing reagents allows for the direct detection of reducing compounds in chromatograms. As a result, the compounds exhibiting the capacity of scavenging ABTS/DPPH radicals can be observed as a bleached band on a green or purple background, respectively. This approach is particularly beneficial in the case of natural extracts or complex matrices where multiple compounds can contribute to the antioxidant activity [36]. The antioxidant profiles of LV extracts revealed after derivatization with both radicals had a similar pattern (Figure 2B,C). The similarity between the profiles of antioxidants detected by TLC-DPPH and TLC-ABTS was also observed in the case of red, rose, and white wines [37]. In the case of these studies, as in the presented analysis of LV extracts, the use of the ABTS radical ensured higher sensitivity of detection as already noted by Soler-Rivas et al. (2000) [38], despite ABTS radical being less stable on the layer than DPPH. In the case of LV extracts studied, the vanillin reagent revealed the presence of several different compounds (Figure 2A), while ABTS and DPPH radicals enabled to determine which of them had antioxidant activity (Figure 2B,C). Based on the obtained profiles, 10 main antioxidants (**I**–**X**) were detected, three of which (**I**, **II**, **VIII**) had the same Rf parameters as the reference compounds used, i.e., ECH (Rf = 0.14), RUT (Rf = 0.29) and OLE (Rf = 0.74), respectively. The intensity of radical discoloration on the plate is positively correlated with the concentration and/or activity of the detected antioxidant, which allows for a simple assessment of extracts as the potential source of redox-active compounds. Extracts from ripe and post-season fruits (RF, PF) contained significantly fewer antioxidants compared to other extracts. Moreover, the ripe fruit extract (RF) was selected for further research because it most likely contained two additional antioxidants that were not found in the leaves (**III**, **V**). 

The (LE) and (RF) extracts were again subjected to the HPTLC analysis for more detailed characterization. Using the TLC-MS interface, the appropriate zones corresponding to the bands of antioxidants in chromatograms were isolated from plates and analysed by HRMS. Figure 2D shows the profile of exemplary extract with gel-free areas remaining after elution of selected zones containing antioxidants intended for identification. The tentative identification of these individual antioxidants (Table 1) was performed based on monoisotopic negative ion [M-H]^−^ mass and fragmentation pattern by comparison with spectroscopic data for standards or compounds previously described in the literature. Additionally, UV-Vis spectra recorded during the analysis of non-derivatized chromatograms with a TLC scanner were also used to identify antioxidants. All detected antioxidants turned out to be phenolic compounds and were classified into three groups: phenylethanoids (4 compounds), iridoids (4 compounds), and flavonoids (2 compounds). 

Phenylethanoids (PE) are one of the most important classes of compounds in the olive family, known for many medicinal properties as well as antioxidant activity [39]. According to Yang et al. (2009) [40], redox properties of phenylethanoid glycosides are related to the number and the location of phenolic hydroxyls, steric hindrance, and also to 2-acetyl in the middle of glucopyranose. As suspected previously, the compound with Rf = 0.14 (**I**) was identified as echinacoside by comparison with standard, which was confirmed by the presence of a characteristic pseudomolecular ion at *m*/*z* 785 and fragment at *m*/*z* 623 formed by the loss of caffeic acid moiety. The other compounds from the PE group were verbascoside (**III**), hydroxytyrosol glucoside (**IV**), and salidroside (**IX**) with pseudomolecular ions at *m*/*z* 623, 315, and 299, respectively. These compounds have been previously identified in plants of the genus *Ligustrum* [41]. 

Another group of detected antioxidants are iridoids (IR), especially secoiridoids of the oleoside type, which constitute a characteristic group of secondary metabolites of *Oleaceae*. One of them (**V**), after derivatization with vanillin reagent, gained a characteristic purple color, which, according to Do et al., 2021 [35] indicates the presence of nuzhenide. The quasi-molecular ion at *m*/*z* 685 confirmed the presence of this iridoid, however antioxidant activity of this compound was relatively low. The strongest antioxidant effect among other secoiridoids is attributed to oleuropein mainly due to the presence of hydroxyl groups (particularly the 1,2-dihydroxybenzene moiety) in its chemical structure. These hydroxyl groups could donate hydrogen to prevent oxidation [42]. In LV extracts studied, compound **VIII** with Rf at 0.74 was identified as oleuropein, based on the characteristic pseudomolecular ion at *m*/*z* 539 and fragment ions at *m*/*z* 377, 307, and 275, which were in line with those of a standard. Another secoiridoid found at Rf = 0.59 (**VII**) appeared as a pseudomolecular ion at *m*/*z* 555, which was 16 Da greater than this typical for oleuropein. Thus, it suggested the presence of an additional hydroxyl group, therefore compound **VII** was assigned as 10-hydroxyoleuropein. Finally, the compound that was detected in the band at Rf = 0.88 (**X**) had a parent ion smaller than that of compound **VII** by 162 Da, suggesting glucose moiety loss. Therefore, compound **X** was identified as 10-hydroxyoleuropein aglycon. 

The third group of redox-active compounds detected in LV extracts were flavonoids, which are widely considered to be powerful antioxidant agents. Only two antioxidants were assigned to this group, namely compound **II** and **VI** with Rf at 0.29 and 0.53, respectively. Compound **II** with pseudomolecular ion at *m*/*z* 609 and specific MS/MS fragments at *m*/*z* 300 and 301 were assigned as quercetin-*O*-rutinoside (rutin). The compound **VI** with precursor ion at *m*/*z* 447 was identified as luteolin-*O*-glucoside based on the presence of characteristic MS2 fragment at *m*/*z* 285. The presence of both compounds was also earlier confirmed in the case of *Ligustrum lucidum* fruit extracts [41].

### 2.2. Separation and Identification of Antioxidants by HPLC-MS

**Figure 3:**
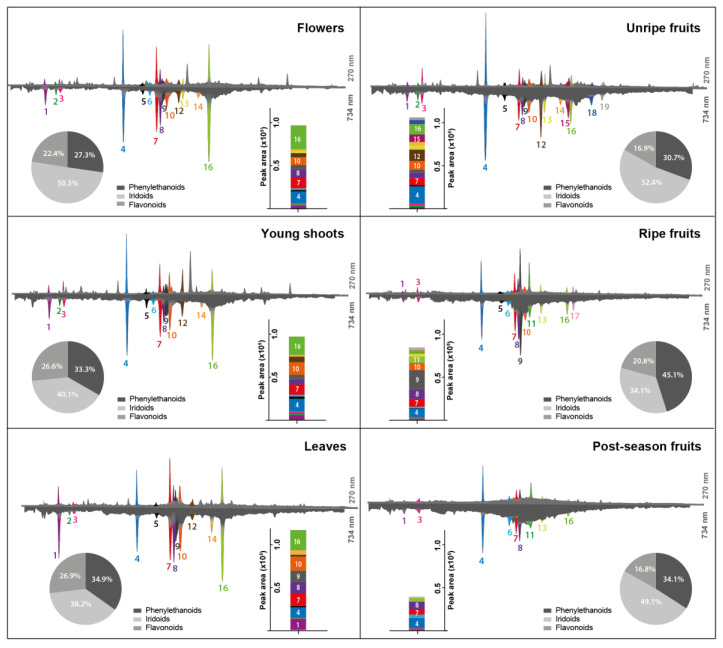
HPLC chromatograms of *Ligustrum vulgare* extracts (top chromatograms at 270 nm) along with profiles of antioxidants detected online with ABTS reagent (bottom chromatograms at 734 nm). The antioxidant activity of samples (bar graphs) was calculated as the sum of areas under the negative peaks of detected antioxidants recorded during post-column derivatisation with ABTS reagent. The contribution of the major groups of phytochemicals to the total antioxidant activity was assessed based on the areas of negative peaks and is presented in the form of pie charts. The compound numbers (1–19) correspond to the numbers in Table 2.

The greatest advantage of using the HPLC-DAD system, coupled with post-column derivatization with ABTS reagent, is the ability to reach, in a single analysis such important goals as chromatographic separation, quantitative data, UV-Vis spectra and information on antioxidant activity of sample components. The ABTS reagent was chosen for post-column derivatization, as it is the most stable during gradient elution with acidified mobile phase and does not pose a risk of crystallization in the system, as may be the case with DPPH or Folin–Ciocalteu reagents [12,13]. Figure 3 presents a chromatographic comparison of the composition of *L. vulgare* phytochemicals (traced at 270 nm, top chromatograms) with their antioxidant activity (traced at 734 nm, bottom chromatograms). The negative peaks recorded at 734 nm, the analytical wavelength of ABTS, result from the chemical reaction between the compounds present in the stream of effluent flowing from a chromatographic column with a stream of reagent solution. The reduction of ABTS radical by separated reductants present in the samples leads to a shift in its UV-Vis absorption spectrum that can serve as a quantitative measure of the antioxidative activity of analytes. 

Among all detected substances, 19 exhibited antiradical activity towards ABTS. The most diverse as regards antioxidants was the extract from unripe fruits, containing 16 compounds with antiradical properties. Compounds **1**, **3**, **4**, **7**, **8,** and **16** were present in all tested extracts. Compound **17** was characteristic for ripe fruits, while compound **11** was present in ripe, but also in post-season fruits. The total antioxidant activities of LV extracts were presented in the form of bar graphs as the sum of negative peak areas. As can be seen, the antioxidant potential was different for each morphological part of the common privet. In general, as the fruit ripeness increased, the antioxidant activity decreased. The antioxidant activity of the leaf extracts was the highest and it was approximately more than twice greater than that of extracts obtained from post-season fruits. 

Because of the use of the same chromatographic separation conditions in LC-HRMS analysis as in the case of antioxidant profiling, it was possible to identify individual compounds responsible for the reduction of ABTS radicals. In this part of the study, heated electrospray ionization (HESI) in negative ion mode was employed, due to the better sensitivity towards corresponding signals. The putative identification of antioxidants from LV extracts presented in Table 2 was based on UV spectral data, molecular masses, and fragmentation patterns obtained by Q-Orbitrap-MS analysis, and comparisons with data from previous studies on the *Oleaceae* family described in literature. Owing to this approach, 7 phenylethanoids, 10 iridoids, and 2 flavonoids could be tentatively characterized.

In the case of the phenylethanoids, four antioxidants were identified: **4**, **9**, **1,** and **3** with the same structure as compounds **I**, **III**, **IV,** and **IX** detected by HPTLC, respectively. Another three (**2**, **5**, **12**) antioxidants from the PE group were additionally detected using the HPLC method. Among simple PE, hydroxytyrosol (**2**) and its glucoside (**1**) were identified based on the characteristic molecular ion at *m*/*z* 153, along with a fragment at *m*/*z* 123 resulting from the loss of CH_3_OH. Additionally, tyrosol glucoside (salidroside, **3**) at *m*/*z* 299 was identified. Moreover, compound **12** with [M-H]^-^ at *m*/*z* 335 showed a fragmentation pattern typical for tyrosol moiety (*m*/*z* 119, 137). However, its structure could not be fully determined as the main molecular ion differs from tyrosol by 198 Da. On the other hand, the MS/MS fragment at *m*/*z* 121 suggests that compound **12** could be assigned as syringic acid. Based on the presence of fragment ions derived from caffeic acid (*m*/*z* 161, 135, 179), two isomers of echinacoside (**4**, **5**) and verbascoside (**9**) were identified. As shown in Figure 3, the main antioxidant present in all extracts tested was echinacoside (**4**) occurring at similar concentration levels, which is consistent with previous observations of HPTLC profiles (Figure 2, compound **I**). 

The use of the HPLC method with post-column derivatization and additional MS analysis enabled the identification of 10 iridoids with antioxidant properties in LV extracts (6 compounds more than by HPTLC method). The four identified iridoids **11**, **8**, **16,** and **14** correspond to the structures of compounds **V**, **VII**, **VIII,** and **X** detected by HPTLC, respectively. The additional six iridoids detected by the HPLC method are compounds with numbers: **6**, **13**,**15**,**17**,**18**,**19**. Compound **16**, with precursor ion at *m*/*z* 539 and characteristic MS fragmentation pattern (*m*/*z* 307, 275, 139) was easily identified as oleuropein. Oleuropein and hydroxytyrosol are compounds mostly responsible for the oxidant stability of extra virgin olive oil [43]. The mass of compound **8,** corresponding to pseudomolecular ion at *m*/*z* 555, was 16 Da greater than this typical for oleuropein. Thus, it suggested the presence of an additional hydroxyl group. The MS/MS fragment at *m*/*z* 307 indicated that the hydroxyl group was connected with oleoside moiety, therefore compound **8** was assigned as 10-hydroxyoleuropein. Compounds **14** and **15** shared fragments characteristic of oleuropein derivatives and fragments at *m*/*z* 393 obtained by the loss of glucose (162 Da) which is why they were identified as 10-hydroxyoleuropein aglycone isomers. Compound **19**, with a molecular ion at *m*/*z* 523 and fragments at *m*/*z* 361, 291, and 259 was tentatively identified as ligstroside. The fragment at *m*/*z* 361 was formed due to the loss of glucose, while the remaining two fragments were formed by the loss of C_4_H_6_O and CH_3_OH. One of the most common components of fruits from the *L. lucidum* tree, which is closely related to *L. vulgare*, is nuzhenide and its derivatives [44], with typical fragments at *m*/*z* 453, 299 (salidroside) and 223. These fragments and precursor ions at *m*/*z* 685 were detected in the case of compound **11**. Similarly, compound **6** with [M-H]^−^ at *m*/*z* 701 and specific hydroxytyrosol glucoside fragment at *m*/*z* 315 was identified as neonuzhenide, which is a nuzhenide derivative. Compound **13** showed precursor ions at *m*/*z* 1171 and a fragmentation pattern similar to echinacoside (*m*/*z* 785, 623). Another fragment at *m*/*z* 1009 formed by the loss of glucose (162 Da), followed by the loss of oleoside 11-methyl ester moiety (−223 Da) suggested that compound **13** was oleoechinacoside. Compound **17** yielded the base peak at *m*/*z* 1009, while the formation of fragments at *m*/*z* 847 and 623, can be attributed to the loss of hexose (162 Da) and oleoside (386 Da), respectively. These data are in line with those reported for oleoacteoside [45]. 

In the case of the flavonoid group, the same two antioxidants were detected using both HPLC (compounds **7** and **10**) and HPTLC (compounds **II** and **VI**). The precursor ion at *m*/*z* 609 of compound **7**, which produced a daughter ion at *m*/*z* 301, was identified as quercetin-*O*-rutinoside. However, a typical fragment at *m*/*z* 285 suggested that compound **10** with pseudomolecular ion at *m*/*z* 447 was an *O*-glycosidic form of luteolin. 

Identification of individual compounds makes it possible to determine the percentage contribution of each chemical group to the antioxidant capacity of the sample based on the areas of negative peaks. The contribution of phenylethanoids, iridoids, and flavonoids to the total antioxidant activity of the tested LV extracts is presented in the form of pie charts in Figure 3. Compared to the other groups, iridoids were mainly responsible for the antioxidant activity in all studied extracts except for the ripe fruits, in which phenylethanoids dominated with verbascoside at the forefront. Flavonoids were responsible only for approximately 25% of the total antioxidant activity exhibited by studied *L. vulgare* extracts.

## 3. Materials and Methods

### 3.1. Chemicals and Reagents 

Pure p.a. ethanol, methanol, and dichloromethane were purchased from POCH (Gliwice, Poland), and MS-grade acetonitrile and formic acid from Merck (Darmstadt, Germany). Water was purified using a QPLUS185 system from Millipore (Bedford, MA, USA). Vanillin, 2,2′-azinobis (ethyl-2,3-dihydrobenzothiazoline-6-sulphonic acid) diammonium salt (ABTS), 2,2-diphenyl-1-picrylhydrazyl (DPPH), oleuropein (OLE), echinacoside (ECH) and rutin (RUT) were purchased from Sigma–Aldrich (St. Louis, MO, USA).

### 3.2. Plant Extracts Preparations

Different morphological parts of *Ligustrum vulgare* L. (LV) were collected at different stages of the growing season in Gdansk (the Gdansk University of Technology campus, Gdańsk, Poland). At the earliest, in spring, the fruits from the previous season that remained on the bush after winter (Postseason fruits—PF) and young shoots (YS) were harvested (May 2022). Then, a month later, the flowers (FL) were collected (June 2022). The green, unripe fruits (UF) were harvested in July, while the dark, ripe fruits (RF) were harvested in September (2022). The leaves (LE) were also collected in September. The plant samples were authenticated by Maura Zaworska from the Department of Environmental Design at the Gdansk University of Technology who specializes in issuing botanical expert opinions. The collected plant material was lyophilized and ground. The freeze-dried powder was mixed with 70% ethanol solution (100 mg/mL) and placed in an ultrasonic bath (POLSONIC, Warsaw, Poland) for 15 min at 25 °C. The samples were centrifuged (3000 rpm, 15 min) and clear supernatants were collected.

### 3.3. Standards and Reagents Preparation

To prepare the oleuropein, echinacoside, and rutin standard solutions, 1 mg of the respective compounds were dissolved in 1 mL of ethanol. The vanillin derivatization reagent was prepared by dissolving 1 g of vanillin in 100 mL of ethanol, followed by the dropwise addition of 2 mL of concentrated sulfuric acid. The DPPH radical solution was prepared in methanol (5 mmol/L) immediately before the experiments and kept in a lightproof container. ABTS was dissolved in aqueous Na_2_S_2_O_8_ (2.45 mmol/L) to obtain a concentration of 7 mmol/L and left in the dark at ambient temperature for 24 h. The obtained stock solution of ABTS was dissolved with methanol to concentrations of 30% (*v*/*v*) for HPTLC and HPLC antioxidant profiling, respectively.

### 3.4. Antioxidants Profiling by HPTLC 

Glass pre-coated silica gel HPTLC plates (20 × 10 cm, F254) were purchased from Merck (Darmstadt, Germany). Samples of LV extracts were processed using a CAMAG HPTLC system (Muttenz, Switzerland) equipped with an automatic ATS4-TLC sampler, an ADC2-TLC developer, a TLC visualizer 2, a TLC scanner 4, a Derivatizer and a visionCATS software v. 2.5. Different volumes of the LV extracts were applied on the plate and the best patterns were produced from 1 μL. Thus, this volume of each sample was spotted as 8 mm bands. For the chromatographic analysis, six samples of LV extracts and three standard solutions were spotted on the plate leaving a 12 mm distance between bands. The chromatographic separation was performed with the use of conditions described by Czerwinska et al. (2015) [33] with slight modification. The mixture of dichloromethane-methanol-formic acid-water (80:25:3:2.5, *v*/*v*/*v*/*v*) was used as a mobile phase. The conditions of development were as follows: tank saturation time 20 min, plate preconditioning time 10 min, relative humidity 33%, plate drying time 10 min, and migration distance 85 mm. After drying, the developed HPTLC plates were photographed under white light, at 254 nm and 366 nm. 

After documentation of the initial chromatographic results, the HPTLC plates were sprayed with 2 mL of the three different derivatization reagents by automatic spraying device. One of the plates was sprayed with vanillin reagent and heated for 3 min at 100 °C, the other two were derivatized with a methanolic solution of the ABTS reagent and DPPH reagent and incubated for 15 min. After cooling and drying the plates were reanalyzed under transmission white light.

### 3.5. Antioxidants Profiling by HPLC

Antioxidant profiling was performed with the use of post-column derivatization with ABTS reagent according to the method described earlier [12,13] with slight modification. To obtain antioxidant profiles of LV extracts, the HPLC-DAD system (1200 series, Agilent Technologies, Wilmington, DE, USA) coupled with the Pinnacle PCX Derivatization Instrument (Pickering Laboratories Inc., Mountain View, CA, USA) was used. Chromatographic separations were conducted on a Kinetex^®^ column (150 × 4.6 mm, 5 μm, Phenomenex, Torrance, CA, USA). The mobile phase consisted of water (solvent A) and acetonitrile (solvent B) both acidified with formic acid 0.1% (*v*/*v*). The flow rate was set at 0.8 mL/min and the injection volume of all samples was 2 μL. The gradient started at 10% B and then increased to 35% B within 25 min, then reached 100% B in 30 min. The column was conditioned with the initial mobile phase for a 7 min period. Absorbance spectra were recorded in DAD between 190 and 700 nm every 2 s with a bandwidth of 4 nm, while the chromatograms were monitored at 236, 270, 325, 360, and 525 nm. 

The eluate leaving the DAD detector was mixed with a methanolic ABTS solution stream (0.1 mL/min) and directed to the reaction loop of the derivatization instrument (PTFE, 1 mL, 130 °C). Then, the eluate stream was led further to the multiple-wavelength detector (MWD 1200 series, Agilent Technologies, Wilmington, DE, USA), where the reduction of ABTS radicals by extract components was monitored at 734 nm.

### 3.6. Identification of Antioxidants

#### 3.6.1. Antioxidants Detected by HPTLC

The selected LV extracts (LE and RF) were applied to the plate four times. The separation conditions were the same as described in Section 3.4. After the plate development and drying, only the first sample track was derivatized with the DPPH reagent. The yellow zones visible on the derivatized track were used for the proper positioning of a round elution head (4 mm) of the TLC-MS interface (CAMAG, Muttenz, Switzerland) on not derivatized tracks. The interface was connected between the HPLC pump and the MS detector. The eluent flow was generated using an Agilent Technologies 1200 series HPLC system. The acetonitrile acidified with formic acid (0.1% *v*/*v*) was used as a mobile phase with a flow rate of 0.2 mL/min. Each selected zone was eluted for 30 s from the plate and transferred into the MS detector operating under the conditions described in Section 3.6.3.

#### 3.6.2. Antioxidants Detected by HPLC 

The LV extracts were reanalyzed at the same separation conditions as described in Section 3.5. but using a different chromatographic system UltiMate 3000 UPLC (Thermo Scientific Dionex, Waltham, MA, USA) equipped with DAD and MS detector. The identification of selected antioxidants was performed based on UV-Vis, MS, and MS2 spectra. The operating conditions of the MS detector are described in Section 3.6.3.

#### 3.6.3. Q-Orbitrap HRMS Analysis

The eluate from the TLC-MS interface or HPLC was directed to a high-resolution Thermo Q-Exactive^TM^ Focus quadrupole-Orbitrap mass spectrometer (Thermo, Bremen, Germany) with a heated electrospray ionization source (HESI II). The HESI parameters in negative polarity mode were as follows: the flow rate of sheath gas, auxiliary gas, and sweep gas was set to 35 arb, 15 arb, and 3 arb, respectively; the spray voltage was 2.5 kV and S-lens RF level was 50; capillary and heater temperature were 350 °C and 300 °C, respectively. The mass range for the full MS scan was 120–1200 *m*/*z* with a resolution of 70,000 FWHM, AGC target at 2 × 10^5^ and max inject time of 100 ms. The parameters of the data-dependent MS2 were as follows: resolution, 17,500; isolation window, 3.0 *m*/*z*; normalized collision energy, 30; AGC target, 1 × 10^6^; max IT, auto.

The raw data from high-resolution mass spectrometry were elaborated with Compound Discoverer (v. 2.1, Thermo, Waltham, MA, USA). Selected compound identification was based on accurate mass and mass fragmentation pattern spectra against MS-MS spectra of compounds available on a customized database of different classes of phytochemicals created based on literature data and implemented in the software.

## 4. Conclusions

Two methods of profiling and identification of antioxidants, based on post-chromatographic derivatization and MS analyses, were compared in this study. Various extracts of *Ligustrum vulgare* were used as an example of a complex natural source of antioxidants to emphasize the usefulness of these methods for comparative studies.

Our study clearly demonstrates that chromatographic techniques combined with radical scavenging assays and MS can be a powerful tool for both qualitative and quantitative analysis of antioxidants in samples from various plant sources.

The main advantage of HPTLC bioautography is the possibility of using numerous derivatizing reagents, depending on the purpose, while maintaining the same separation conditions. Moreover, this approach allows to perform parallel analyses for many samples, which is useful in the field of quality control, as well as in the search for new natural sources of bioactive compounds. As shown in this study, the differences between morphological parts of *L. vulgare* and various levels of maturity could be easily distinguished by HPTLC profiling and appropriate derivatization. The MS identification of compounds of interest can be performed directly from the TLC plate by using the TLC-MS interface. However, the information on the relative antioxidant capacity of individual compounds is strongly dependent on the band separation. In TLC separation it is difficult to distinguish overlapping bands and spots and therefore quantitative assessment of antioxidant activity is not very precise.

In contrast, peaks in HPLC separation can be easily resolved by modifying operational parameters such as mobile phase flow rate or column oven temperature. HPLC-post column derivatization with ABTS allows for a direct link of antiradical activity to a specific compound, thus providing greater sensitivity and resolution than HPTLC-ABTS or HPTLC-DPPH assays. Additionally, by summing the areas under the negative peaks, a semi-quantitative determination of the total antioxidant activity can be obtained. However, in this method, the choice of the derivatization reagent is limited and depends on its stability in the changing composition of the mobile phase during gradient elution, the use of which is necessary to separate the components of a complex mixture. Moreover, to perform MS analysis, additional chromatographic separation of samples is required, which further extends the analysis time.

Nonetheless, both methods allowed to obtaining of profiles and identification of antioxidants present in *L. vulgare* extracts. As the antioxidant activity depended on the part and maturity of the plant sample, *L. vulgare* leaves were found to as the best source of antioxidants. Detected antioxidants were predominantly from the group of iridoids and phenylethanoids, especially oleuropein and echinacoside.

## Figures and Tables

**Table 1:** Major antioxidants detected by HPTLC-bioautography and identified by LC-Q-Orbitrap HRMS in *Ligustrum vulgare* extracts (70% ethanol).

No	Rf	λmax	Formula	Theoretical [M-H]^−^	Experimental [M-H]^−^	Δm [ppm]	MS/MS	Compound	Class
I	0.135	250, 330	C_35_H_46_O_20_	785.250425	785.249756	0.90	133.028; 785,250; 161.023; 135.044; 132.020; 623.219; 786.253; 71.012; 123.044	Echinacoside	PE
II	0.289	203, 265, 360	C_27_H_30_O_16_	609.145565	609.145325	0.39	609.145; 271.024; 255.029; 243.029; 300.027; 301.035; 227.034; 151.002; 199.039	Quertecin-*O*-rutinoside	Flav
III	0.384	237, 285, 324	C_29_H_36_O_15_	623.1976	623.197693	−0.15	133.028; 623.198; 161.023; 135.043; 624.201; 132.020; 85.028; 461.166; 71.012	Verbascoside	PE
IV	0.392	242, 283	C_14_H_20_O_8_	315.107995	315.108429	−1.38	315.108; 135.044; 71.012; 89.022; 119.033; 101.023; 123.043; 316.112; 113.023	Hydroxytyrosol glucoside	PE
V	0.495	239, 280	C_31_H_42_O_17_	685.23438	685.234131	0.36	453.140; 68.997; 59.012; 101.023; 421.151; 71.012; 299.113; 119.049; 523.182	Nuzhenide	IR
VI	0.531	239, 271, 349	C_21_H_20_O_11_	447.09274	447.092957	−0.48	447.093; 285.040; 284.032; 133.028; 448.096; 63.022; 107.012; 151.002; 227.034	Luteolin-*O*-glucoside	Flav
VII	0.587	236, 280	C_25_H_32_O_14_	555.171385	555.171631	−0.44	555.171; 93.033; 71.012; 523.145; 85.028; 89.023; 111.007; 135.044; 273.077	10-Hydroxyoleuropein	IR
VIII	0.739	242, 281	C_25_H_32_O_13_	539.17647	539.176514	−0.08	59.012; 275.093; 307.082; 377.124; 95.049; 71.012; 121.028; 68.997; 111.007	Oleuropein	IR
IX	0.810	230, 240, 278	C_14_H_20_O_7_	299.11308	299.113708	−2.10	-	Salidroside	PE
X	0.887	227, 277	C_19_H_22_O_9_	393.11856	393.118988	−1.09	111.007; 93.033; 273.077; 111.044; 68.997; 275.056; 139.002; 101.023; 307.082	10-Hydroxyoleuropein aglycone	IR

Classes: IR, iridoids; Flav, flavonoids; PE, phenylethanoids.

**Table 2:** Major antioxidants detected by HPLC-ABTS and identified by LC-Q-Orbitrap HRMS in *Ligustrum vulgare* extracts (70% ethanol).

No	Rt [min]	λmax	Formula	Theoretical [M-H]^−^	Experimental [M-H]^−^	Δm [ppm]	MS/MS	Compound	Class
1	3.3	196, 220, 280	C_14_H_20_O_8_	315.10799	315.10873	−2.3	123.044; 59.012; 153.055; 89.023; 101.023; 113.023	Hydroxytyrosol glucoside	PE
2	4.2	196, 220, 280	C_8_H_10_O_3_	153.05517	153.05467	3.2	123.044; 137.023	Hydroxytyrosol	PE
3	4.6	194, 222, 275	C_14_H_20_O_7_	299.11308	299.11368	−1.9	59.012; 119.049; 89.023; 101.023; 113.023; 119.035	Salidroside	PE
4	10.2	195, 220, 330	C_35_H_46_O_20_	785.25042	785.25128	−1.1	785.251; 623.220; 161.023; 162.027; 179.035	Echinacoside (isomer 1)	PE
5	11.9	197, 220, 330	C_35_H_46_O_20_	785.25042	785.25128	−1.1	785.251; 623.220; 161.023; 162.027	Echinacoside (isomer 2)	PE
6	12.6	196, 230, 282	C_31_H_42_O_18_	701.22929	701.23016	−1.2	315.109; 469.135; 437.148; 539.177	Neonuzhenide	IR
7	13.2	202, 220, 256, 355	C_27_H_30_O_16_	609.14556	609.14624	−1.1	301.035; 300.027; 178.998; 151.003	Quertecin-*O*-rutinoside	Flav
8	13.5	196, 232, 283	C_25_H_32_O_14_	555.17138	555.17199	−1.1	273.077; 89.023; 137.023; 101.023; 119.034; 181.050; 111.044; 307.082; 275.056	10-Hydroxyoleuropein	IR
9	13.7	198, 222, 286, 335	C_29_H_36_O_15_	623.19760	623.19836	−1.2	161.023; 461.166; 135.043; 179.034; 315.108	Verbascoside	PE
10	14.1	202, 220, 252, 350	C_21_H_20_O_11_	447.09274	447.09354	−1.8	285.040; 284.032; 447.093; 327.051; 297.040; 269.044; 328.051; 133.028	Luteolin-*O*-glucoside	Flav
11	14.6	200, 228, 280	C_31_H_42_O_17_	685.23438	685.23511	−1.1	453.140; 421.151; 299.114; 223.061; 523.182; 119.034	Nuzhenide	IR
12	15.2	200, 228, 280	C_17_H_20_O_7_	335.11308	335.11374	−1.9	107.049; 119.049; 137.059; 121.028; 109.064; 59.012; 69.033; 108.052; 111.044	Tyrosol derivative (syringic acid)	PE
13	15.6	196, 230, 335	C_52_H_68_O_30_	1171.37173	1171.37183	−0.1	785.252; 1009.328; 1010.339; 623.223; 292.968	Oleoechinacoside	IR
14	16.9	200, 225, 282	C_19_H_22_O_9_	393.11856	393.11945	−2.2	111.044; 93.033; 111.007; 139.002; 101.023; 127.039; 137.023; 181.050; 137.060	10-Hydroxyoleuropein aglycone (isomer 1)	IR
15	17.6	200, 222, 282	C_19_H_22_O_9_	393.11856	393.11951	−2.4	111.007; 111.044; 139.002; 93.033; 127.039; 101.023; 113.023; 137.023; 85.028	10-Hydroxyoleuropein aglycone (isomer 2)	IR
16	17.7	195, 235, 283	C_25_H_32_O_13_	539.17647	539.17737	−1.7	89.023; 275.093; 95.049; 307.082; 149.023; 59.012; 101.023; 139.039; 119.034	Oleuropein	IR
17	18.5	196, 222, 335	C_46_H_58_O_25_	1009.31890	1009.31946	−0.6	847.276; 623.197; 665.215; 161.024; 815.241; 461.169; 252.460	Oleoacteoside	IR
18	19.8	200, 225, 280	C_21_H_28_O_10_	439.16042	439.16107	−1.5	111.007; 111.044; 137.060; 135.044; 179.034; 139.002; 211.061; 93.033; 275.056	10-Hydroxyoleuropein aglycone derivative	IR
19	20.7	194, 225, 280	C_25_H_32_O_12_	523.18155	523.18237	−1.6	291.087; 101.023; 259.097; 127.039; 139.039; 111.007; 89.023; 69.033	Ligstroside	IR

Classes: IR, iridoids; Flav, flavonoids; PE, phenylethanoids.

## Data Availability

Data is contained within the article.
